# Eradication of Vancomycin-Resistant Enterococci by Combining Phage and Vancomycin

**DOI:** 10.3390/v11100954

**Published:** 2019-10-16

**Authors:** Mor Shlezinger, Shunit Coppenhagen-Glazer, Daniel Gelman, Nurit Beyth, Ronen Hazan

**Affiliations:** 1 Department of Prosthodontics, Hadassah School of Dental Medicine, Hebrew University, Jerusalem 91120, Israel; mor.haramaty@gmail.com (M.S.); nuritb@ekmd.huji.ac.il (N.B.); 2 Faculty of Dental Sciences, Hadassah School of Dental Medicine, Hebrew University, Jerusalem 91120, Israel; tinushy@yahoo.com (S.C.-G.); dnlgelman@gmail.com (D.G.)

**Keywords:** phage therapy, *Enterococcus faecalis* (VRE), vancomycin, phage EFLK1, phage-antibiotic synergy (PAS)

## Abstract

Currently, effective options are needed to fight vancomycin-resistant *Enterococcus faecalis* (VRE). The present study shows that combinations of phage and vancomycin are highly efficient against VRE, despite being resistant to the antibiotic. Vancomycin-phage EFLK1 (anti-*E. faecalis* phage) synergy was assessed against VRE planktonic and biofilm cultures. The effect of the combined treatment on VRE biofilms was determined by evaluating the viable counts and biomass and then visualized using scanning electron microscopy (SEM). The cell wall peptidoglycan was stained after phage treatment, visualized by confocal microscopy and quantified by fluorescence activated cell sorting (FACS) analysis. The combined treatment was synergistically effective compared to treatment with phage or antibiotic alone, both in planktonic and biofilm cultures. Confocal microscopy and FACS analysis showed that fluorescence intensity of phage-treated bacteria increased eight-fold, suggesting a change in the peptidoglycan of the cell wall. Our results indicate that with combined treatment, VRE strains are not more problematic than sensitive strains and thus give hope in the continuous struggle against the current emergence of multidrug resistant pathogens.

## 1. Introduction

Antibiotic resistance became a major problem soon after penicillin and sulfonamides were introduced to the world of medicine. In the last few decades, the prevalence of pathogens resistant to antimicrobial agents has increased alarmingly. In the USA, 50–60% of nosocomial infections are caused by antibiotic-resistant bacteria [1]. Gram-positive enterococci are an example of bacteria with extreme resistance. Although enterococci are considered part of the normal microbiota of the human gastrointestinal and genitourinary tracts, they often cause infections that are difficult to eradicate and may lead to mortality rates of 19–48% [2]. Most human enterococcal infections are caused by *Enterococcus faecalis* (*E. faecalis)* and *Enterococcus faecium* [3]. These pathogens are the third and fourth most prevalent nosocomial pathogens worldwide, respectively [1]. Among enterococci, *E. faecalis* causes 80–90% of infections [1]. Vancomycin-resistant enterococci (VRE) first appeared in Europe in 1986 [4] and are considered major causes of nosocomial infections [4]. The problematic nature of these infections is due to enterococci being intrinsically resistant to many antimicrobial agents with only a few antibiotics active against them [5].

A possible solution for targeting resistant bacteria is to use bacterial viruses, known as bacteriophages (phages). Billions of years of evolution [6] have shaped lytic phages to become efficient bacterial killers [7] by causing the infected bacteria to lyse. The number of phage variants for each bacterial species is extremely high because phages tend to readily mutate [6,8], thereby offering many treatment possibilities and combinations. Some phages have advantages over antibiotics in various aspects. Phages can efficiently destroy some biofilms and coevolve with their host, and their specificity leaves the normal microbiota of patients intact [9,10]. Phages multiply at the sites of infection and disappear after their target bacteria lyse, in which case they no longer have the ability to propagate and are removed by the reticuloendothelial host defense system [11]. Furthermore, most phages are nontoxic [12,13], while some induce a limited immune response [14,15]; Therefore, these varied properties may be advantageous when targeting multidrug-resistant bacteria and biofilm inhabitants.

Phage EFLK1, a phage capable of infecting *E. faecalis* (named after its founder Leron Khalifa), was previously isolated and reported, showing a potent effect against VRE [10,16], even in cases of resistance to another *E. faecalis* phage such as EFDG1 [10]. Specifically, phage EFLK1 DNA was found to have a circular genome of 130,952 bp, assembled from 209 putative coding sequences, lacking tRNA genes [16]. A cluster of DNA replication components, including DNA helicases, DNA polymerases, DNA maturase, DNA exonucleases, resolvase, primase and transcription genes, RNA polymerase and sigma factors, were reported [16]. Integrases and ParA/B (genes for plasmid inheritance in prophages), lysogenic marker genes [17], were not present. Phage EFLK1 was found to be a member of the *Spounavirinae* family, which is a *Myoviridae* phage subfamily [16]. *Myoviridae* phages are predominantly recognized as lytic phages [18].

In most recent cases of phage therapy, including one run by our group [19], phages were given in conjugation with antibiotics. The combined treatment increases the number of possibilities to treat resistant bacteria [20] based on the evolutionary understanding that two selective pressures may be more effective than either alone. Phages have the potential to be a successful complement to antibiotics due to the differences in their mechanisms of action [21]. When the effect of an interaction between stressors that are working in the same direction is greater than the sum, it is called synergistic [22]. Phage-antibiotic synergy (PAS) has been previously described in several species, including *E. faecalis* [23] *Escherichia coli* [24,25], *Klebsiella pneumonia* [26], *Burkholderia cenocepacia* [27], *Staphylococcus* [28,29,30] and *Pseudomonas aeruginosa* [30,31,32]. Moreover, the PAS effect was reported in the *Burkholderia cepacia* complex, which possesses high levels of innate antimicrobial resistance [27]. Kamal et al. suggested that antibiotics can be combined with phages to stimulate increased phage production and/or activity and thus improve the efficacy of bacterial killing [27]. Gelman et al. showed that a dual treatment of phage cocktail and antibiotics had the best clinical effect on severe septic peritonitis caused by *E. faecalis* in a mouse model [23]. Consequently, combined treatment with phage and antibiotic increases the possibilities for combatting resistant pathogens.

In this study, we tested the combined effect of a phage and an antibiotic to which the bacteria are resistant. We simulated a situation where a sensitive *E. faecalis* was treated by vancomycin, and a VRE mutant emerged. The question we asked was whether vancomycin treatment should be topped or continued in such cases. Intriguingly, we found that vancomycin synergizes the effect of the phage even on the VRE strain. Combined treatment with phages and vancomycin had an antibacterial synergistic effect against vancomycin-resistant *E. faecalis* (VRE) with a greater impact than each treatment alone. Such combinations may determine the future evolution of antibiotic-resistant pathogen treatment.

## 2. Materials and Methods

### 2.1. Bacterial and Phage Strains

Vancomycin-resistant *E. faecalis* V583 (ATCC 700802, GenBank AE016830.1), clinically isolated from human blood [33,34], served as the test organism. 

For each experiment, an aliquot of frozen bacteria was thawed and transferred to a test tube containing brain heart infusion (BHI) medium (Difco Laboratories, Detroit, MI, USA) and grown for 24 h at 37 °C under aerobic conditions. Then, the cultures were diluted 1:1000 into a new tube with fresh BHI medium and grown for 2 h to mid-log phase (optical density OD_600 nm_ = 0.5). Additional bacterial strains used for screening were vancomycin-sensitive *E. faecalis* Aef01, *E. faecalis* Aef03 and *E. faecalis* Aef05, clinical isolates from The Hadassah Medical Center, Jerusalem, Israel. The phage that was tested here was EFLK1, a member of the *Spounavirinae* subfamily that was previously isolated by our lab and showed a potent effect against *E. faecalis* V583 [10,16]. The phage was purified by picking a single plaque, removing it from the agar plate, and growing it with the bacteria.

### 2.2. Materials

Unless otherwise stated, all materials were purchased from Sigma-Aldrich (St. Louis, MO, USA). Vancomycin hydrochloride (*Streptomyces orientalis*, CAS: 1404-93-9, Sigma-Aldrich) was purchased from Sigma-Aldrich, Israel (Rehovot).

### 2.3. Determination of Vancomycin Minimal Inhibitory Concentration (MIC)

A stock solution of 2 mg/mL vancomycin hydrochloride was prepared in BHI broth. Serial dilutions were made in 96-well microtiter plate (Thermo Fisher Scientific, Roskilde, Denmark) ranged from 0–0.25 mg/mL: 0.25, 0.125, 0.062, 0.031, 0.015, 0.007, 0.003 and 0.001 mg/mL, in a final volume of 100 µL. Exponential phase bacteria (10^5^ colony forming units (CFU)/mL) were added to the wells in a final volume of 200 µL, and the culture growth kinetics were recorded immediately after treatment for 24 h at 37 °C shaking for 5 s every 20 min in a 96-well plate reader (Synergy; BioTek, Winooski, VT, USA) at optical density OD_600 nm_. The MIC was defined as the lowest concentration of antimicrobial that inhibited the bacterial growth.

### 2.4. Host-Range Specificity Tests

The activities of phage EFLK1, vancomycin and combined treatment of phage EFLK1 and vancomycin were screened against clinical isolates of vancomycin-sensitive *E. faecalis*; *E. faecalis* Aef01, *E. faecalis* Aef03 and *E. faecalis* Aef05, from the infectious disease unit of Hadassah Hospital (Table 1). Bacterial growth kinetics were monitored using a 96-well plate reader.

### 2.5. Assessment of Phage and Antibiotic Lytic Activity in Planktonic Cultures

To investigate whether PAS occurs between phage EFLK1 [16] and vancomycin hydrochloride, a series of concentrations of each of the two antimicrobials were prepared and tested alone or in combination in 96-well microtiter plates. Phage EFLK1 ranged from 0–10^9^ plaque forming units (PFU)/well: 1 × 10^9^ PFU/well, 5 × 10^8^ PFU/well, 2.5 × 10^8^ PFU/well, 1.2 × 10^8^ PFU/well, 6.2 × 10^7^ PFU/well, 3.1 × 10^7^ PFU/well, and vancomycin hydrochloride ranged from 0–0.25 mg/mL: 0.25, 0.125, 0.062, 0.031, 0.015, 0.007, 0.003 and 0.001 mg/mL (for a schematic presentation, see Figure S1). Exponential phase *E. faecalis* V583 (10^5^ colony forming units (CFU)/mL) were added to each plate, and the culture growth kinetics were recorded immediately after treatment for 24 h at 37 °C shaking for 5 s every 20 min in a 96-well plate reader (Synergy; BioTek, Winooski, VT, USA) at optical density OD_600_. Viable counts (CFU/mL) were evaluated after 24 h. Ten microliters of serial dilutions of the samples were plated on BHI. Colonies were counted after 24 h at 37 °C.

### 2.6. Assessment of Phage Lytic Activity in a Biofilm

*E. faecalis* V583 static biofilms were grown in BHI broth for 72 h in a 96-well microtiter plate at 37 °C, as previously described [35]. To produce a 72-h biofilm, the medium was replaced every 24 h with fresh medium. Phage EFLK1 (10^9^ PFU/well) and vancomycin were serially diluted (in rows and columns, see Figure S1) as described above for the planktonic bacteria and added to the biofilm. After 96 h of incubation at 37 °C, the wells were washed with phosphate-buffered saline (PBS), and the biomass was quantified after crystal violet staining as previously described [36]. Briefly, 200 µL of methanol was added to each well, followed by incubation for 20 min. The methanol was then aspirated, and the wells were air-dried. The biofilms were stained with 200 µL of crystal violet (1%) for 20 min at 20 °C and then washed with water. A 200 µL volume of ethanol was added, the biomass was determined at OD_538_, and the CFU/mL were evaluated. Wells were scraped thoroughly, paying attention to the well edges. Each well content was transferred to 1.5 mL tubes and placed in a sonicating water bath (Bandelin Sonopuls HD 2200, Berlin, Germany) for 5 min to disrupt the biofilm, and 10 µL of serial dilutions of each sample were plated on BHI. Colonies were counted after 24 h at 37 °C.

### 2.7. Scanning Electron Microscopy (SEM)

The biofilm was grown as described above on glass coverslips. Samples were fixed in Karnovsky’s fixative (2% paraformaldehyde, 2.5% glutaraldehyde in 0.1 M cacodylate buffer, pH 7.4) for 4 h at 20 °C, followed by 50% diluted Karnovsky’s fixative (diluted in 0.1 M cacodylate buffer) overnight at 4 °C. The samples were then postfixed in 1% OsO_4_ in 0.1 M cacodylate buffer for 2 h, dehydrated through a graded alcohol series, and placed in a critical point drier (Quorum Technologies, K850 Critical Point Drier Ashford, Kent, UK). After sputtering (Quorum Technologies, SC7620 Spatter coater) with Au/Pd, samples were viewed under SEM (Quanta 200, FEI, Brno, Czech Republic).

### 2.8. Detection of N-Acetylglucosamine in Cell-Wall

To investigate the mechanism of synergistic effect between vancomycin and phage EFLK1, *N*-acetylglucosamine (GlcNAc) was chosen as a target, because of its relativity to vancomycin target d-Ala-d-Ala terminus of peptidoglycan. Wheat germ agglutinin (WGA)-Alexa Fluor 647 conjugate (Invitrogen, Basel, Switzerland) was used for detection of GlcNAc on *E. faecalis* cell wall [37]. Phage EFLK1 was added to planktonic *E. faecalis* V583. After 48 h at 37 °C incubation, the culture was plated on BHI agar plates and incubated for 24 h at 37 °C. A single colony was inserted into a fresh BHI medium and grown for 24 h at 37 °C, the culture was named *EFLK1 survivor (EFLK1s).* Untreated *E. faecalis* V583 served as control. The cultures were diluted 1:1000 into a new tube with fresh BHI medium and grown for 2 h to mid log phase (optical density OD600nm = 0.5). Bacterial cells were harvested by centrifugation and resuspended in phosphate-buffered saline tween (PBST) in a 1:10 ratio (120 mM NaCl, 50 mM phosphate, 0.1% Tween 20, pH 8.0). 100 µL cells and 50 µL of Alexa Fluor 647 WGA solution (0.1 mg/mL) were mixed and incubated for 10 min at 25 °C. *E. faecalis* V583 and *EFLK1s* cells were removed from labeling solution by centrifugation (12,000× *g*, 1 min) and washed twice with PBST buffer. After washing the cells, they were examined by confocal scanning fluorescence microscope (Olympus FV300, Tokyo, Japan) with a ×60 lens. Additionally, a quantification of the stained cells was conducted by using fluorescence activated cell sorting (FACS) analysis. The cells were filtered through a cell strainer (70 µm) and analyzed with Accuri C6 flow cytometry (BD Biosciences, San Jose, CA, USA). 

### 2.9. Statistical Analysis

The results were analyzed as the mean ± standard deviation (STDEV.P function in Excel) in each experimental group. Statistical significance was calculated by a Student’s *t*-test two-tailed unpaired *p* values (significance level: *p* < 0.05, *p* < 0.01).

## 3. Results

### 3.1. Combined Treatment with Vancomycin and Phage EFLK1 Reduced VRE Planktonic Growth

**Figure 1:**
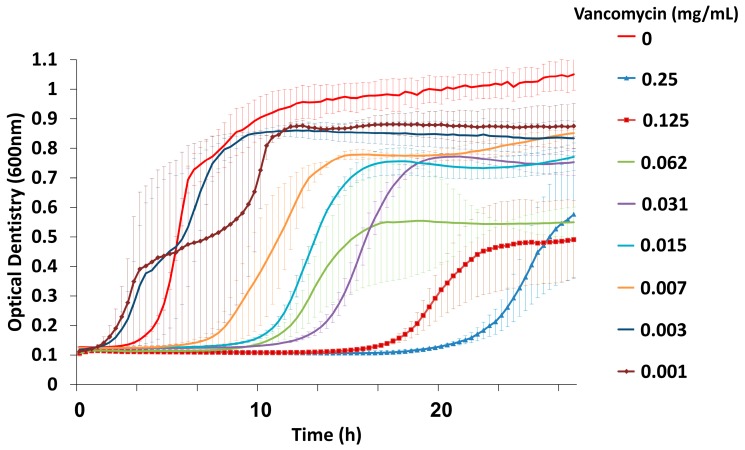
Representative depiction of vancomycin minimal inhibitory concentration (MIC) for *E. faecalis* V583 (VRE) by serial dilutions. The results are mean ± SD based on three independent biological replicates.

The vancomycin minimal inhibitory concentration (MIC) tested in planktonic VRE cells (MIC > 0.25 mg/mL) showed that the highly resistant bacteria exhibited a dose-dependent growth reduction as the antimicrobial effect of the drug was proportional to the given drug dose (Figure 1), as can be seen by the long duration of the lag phase and the decreased final OD after treatments by higher vancomycin concentration. Cells plated on BHI agar showed bacterial growth at all concentrations.

**Figure 2:**
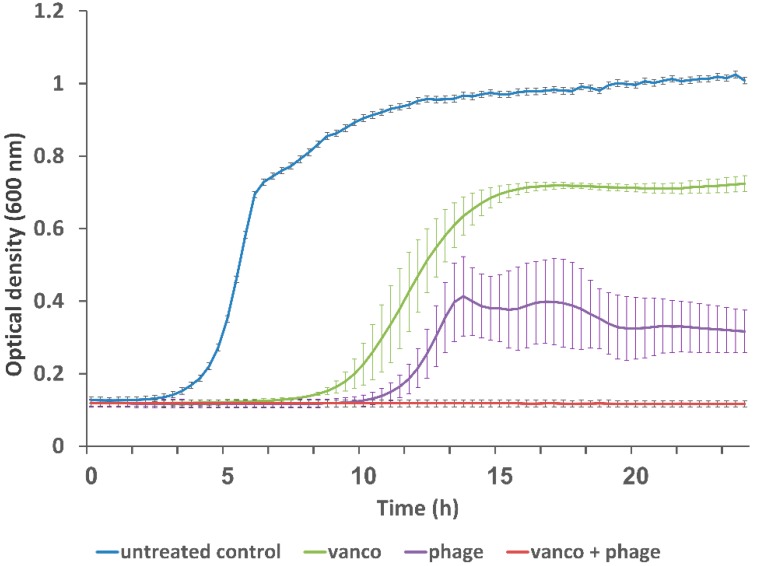
Treatment of planktonic bacteria with 1.2 × 10^8^ plaque forming units (PFU)/well phage EFLK1 (anti-*E. faecalis* phage) combined with 0.015 mg/mL vancomycin reduced VRE growth. Total growth was inhibited in the combination of 0.015 mg vancomycin/mL and 1.2 × 10^8^ PFU/well phage EFLK1 (red). VRE control depicts untreated bacteria showing maximal growth (blue). A longer lag phase was observed after treatment with 0.015 mg/mL vancomycin (green) or phage EFLK1 (1.2 × 10^8^ PFU/well) alone (purple). The combined treatment reduced the bacterial maximum optical density (OD) with better effect than that of the phages or vancomycin alone. The results are mean ± SD based on 3 independent biological replicates.

After examining all the combinations of phage EFLK1 and vancomycin (Figure S2), we decided to focus on the mixture of the lowest treatment concentrations that yielded the best synergistic effect on both planktonic and biofilm bacteria and was within the therapeutic range of vancomycin (<0.031 mg/mL): 0.015 mg/mL vancomycin and 1.2 × 10^8^ PFU/well phage EFLK1. Maximal bacterial growth was observed in the untreated VRE controls, and minimal growth was observed in the combination of 0.015 mg/mL vancomycin and 1.2 × 10^8^ PFU/well phage EFLK1. Treatment with vancomycin alone caused slightly reduced bacterial growth. Phage EFLK1 alone reduced the culture’s turbidity, as reflected in its optical density at 600 nm (OD_600_), although the inhibition was less efficient than when combined with vancomycin (Figure 2).

**Figure 3:**
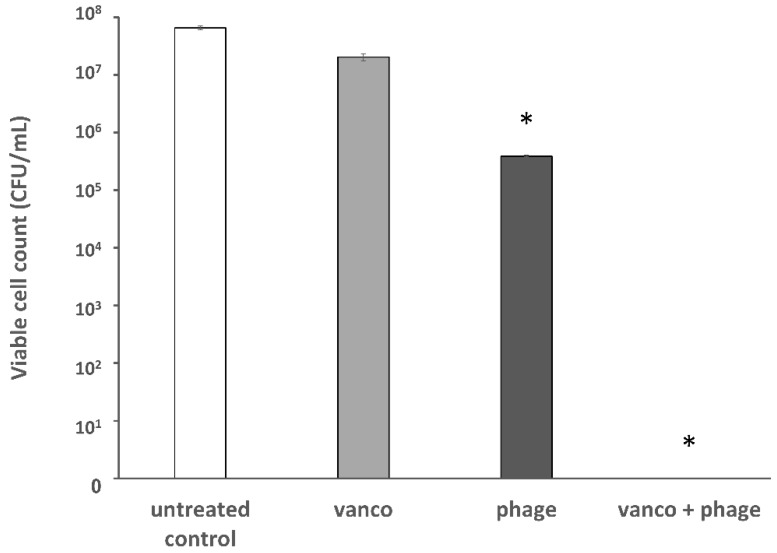
Viable counts of planktonic vancomycin-resistant *E. faecalis* following a combined treatment of phage EFLK1 and vancomycin. The colony forming units (CFU)/mL of VRE treated with 0.015 mg/mL vancomycin combined with phage EFLK1 1.2 × 10^8^ PFU/well is presented. Bacteria were below the limit of detection after treating the cells by combining phage EFLK1 and vancomycin. Bacteria treated only with vancomycin showed survival scores like those of the untreated bacteria; cells treated with phage EFLK1 showed medium survival rates. Combining vancomycin and phage EFLK1 caused seven log reductions in CFU/mL. Light gray = vancomycin-treated bacteria, dark gray = phage EFLK1 treatment, black = phage EFLK1 + vancomycin. Statistically significant (*p* < 0.01) compared to the untreated control. The results are mean ± SD based on three independent biological replicates.

To validate these results, viable cells were counted (Figure 3). Bacterial inhibition (seven logs reduction) was observed after treatment with the combination of phage EFLK1 and vancomycin, an effect that was not observed by vancomycin treatment. Phage EFLK1 treatment reduced two logs of the viable counts.

The infectivity of phage EFLK1, vancomycin and their combined treatment were assessed on several clinically isolated bacteria. Table 1 denotes the details of the tested bacteria, including their antibiotic resistance. Phage EFLK1 was found to be host-specific, infecting *E. faecalis* strains. In *E. faecalis* strains (*E. faecalis* V583, *E. faecalis* Aef01, *E. faecalis* Aef03, *E. faecalis* Aef05), adding phage EFLK1 with vancomycin showed better results than vancomycin alone, reducing the vancomycin MIC.

### 3.2. Combined Treatment of Vancomycin and Phage EFLK1 Reduced 72 h Biofilms

**Figure 4:**
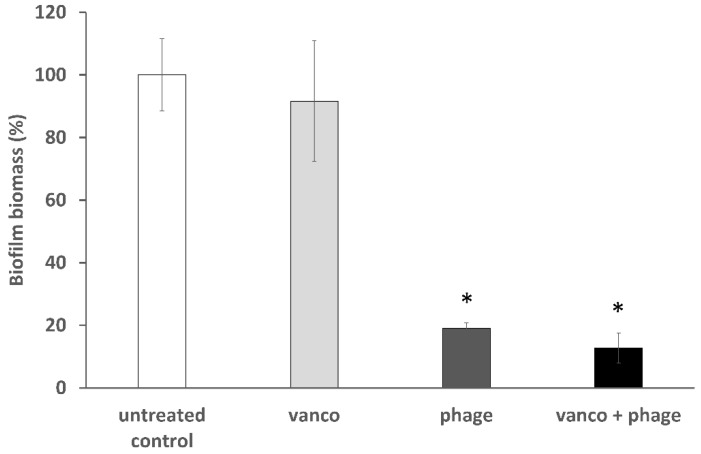
*E. faecalis* biofilm biomass following treatment with phage EFLK1 (1.2 × 10^8^ PFU/well) and vancomycin (0.015 mg/mL). Treatment with combinations of phage EFLK1 and vancomycin significantly decreased bacterial biomass (87% reduction) as evaluated by crystal violet staining. The results are presented as percentages, normalized to the biofilm biomass controls. Light gray = vancomycin-treated bacteria, dark gray = phage EFLK1 treatment served as the control, black = phage EFLK1 + vancomycin treatment. Statistically significant (*p* < 0.05) compared to the untreated control. The results are mean ± SD based on three independent biological replicates.

**Figure 5:**
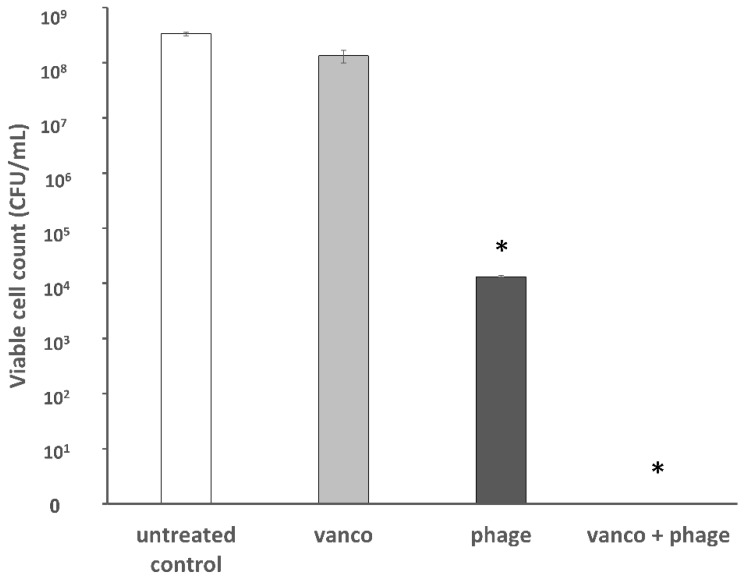
VRE 72-h biofilm viable counts following a combined treatment of phage EFLK1 (1.2 × 10^8^ PFU/well) and vancomycin (0.015 mg/mL). Combining vancomycin and phage EFLK1 caused an eight logs reduction in the CFU/mL of the bacterial biofilm, bacteria were below the limit of detection. Phage EFLK1 caused four log reductions, and vancomycin caused less than a log reduction. Light gray = vancomycin treatment alone, dark gray = phage EFLK1 treatment alone, served as the control, black = phage EFLK1 + vancomycin treatment. Statistically significant (*p* < 0.05) compared to the untreated control. The results are mean ± SD based on three independent biological replicates.

**Figure 6:**
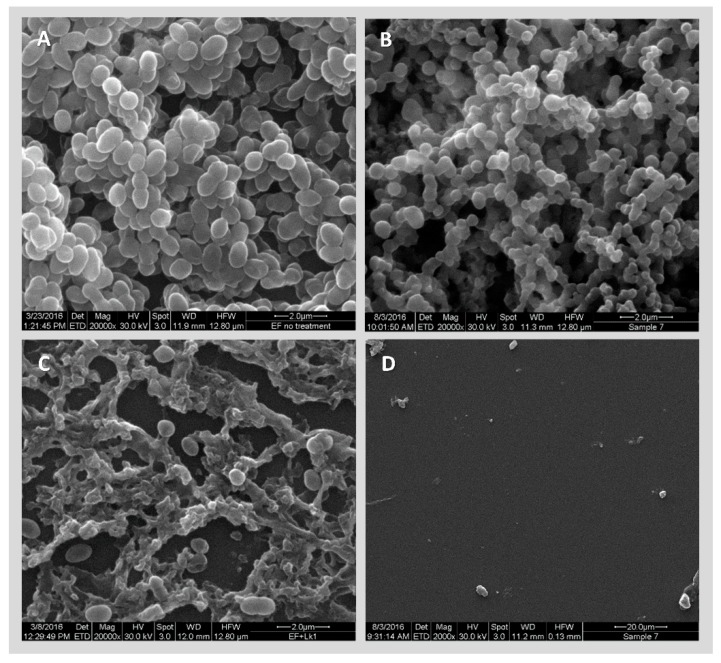
Combinations of phage EFLK1 and vancomycin target VRE. Scanning electron microscopy images (MAG: **A**–**C**: 20,000, **D**: 2000): (**A**) *E. faecalis* 72-h biofilm. (**B**) Biofilm exposed to vancomycin alone, showing no effect. (**C**) *E. faecalis* biofilm exposed to phage EFLK1 showing bacterial lysis, leaving mainly the extracellular matrix. (**D**) *E. faecalis* biofilm exposed to vancomycin and phage EFLK1 showing massive bacterial lysis, degradation and biofilm deformation, leaving almost no trace of biofilm.

Biofilms are one of the most challenging infection modalities to treat [38]. Thus, a combined treatment of phage EFLK1 and vancomycin against *E. faecalis* biofilm was investigated. After examining all the combinations of phage EFLK1 and vancomycin (Figure S3), we decided to focus on the mixture of the lowest concentrations that yielded the best synergistic effect both in planktonic bacteria and in biofilm: 0.015 mg/mL vancomycin and 1.2 × 10^8^ PFU/well phage EFLK1. Consistent with the results for the planktonic cultures, combinations of phage EFLK1 and vancomycin increased killing in the vancomycin-resistant *E. faecalis* biofilm (Figure 4, Figure 5 and Figure 6). Biofilm biomass was assessed by crystal violet staining (Figure 4). Following combined treatment of phage EFLK1 (1.2 × 10^8^ PFU/well) and vancomycin (0.015 mg/mL), the biomass decreased by 87%. Biofilm treated with vancomycin alone showed high biomass levels with only an 8% decrease. Treatment of biofilm with phage EFLK1 alone reduced the biofilm biomass by 81% (Figure 4). There was no high difference between phage treatment alone and phage combined with vancomycin, so we further tested the viable counts in order to eliminate the presence of non-viable debris and matrix remanences. Viable counts of *E. faecalis* biofilm treated with vancomycin combined with phage EFLK1 showed growth reduction to an undetectable level, whereas treatment with vancomycin alone showed less than one log growth reduction, and phage EFLK1 treatment alone caused four log growth reduction (Figure 5).

Finally, SEM was used to visualize the effect of phage EFLK1 and vancomycin on a 72-h VRE biofilm (Figure 6). Exposure to vancomycin alone had little effect on the bacterial cells, reducing the size of the bacteria (Figure 6, panel B), while biofilm exposed only to phage EFLK1 revealed extensive bacterial lysis (Figure 6, panel C), leaving a large amount of cellular debris. As in the planktonic culture, *E. faecalis* biofilm exposed to vancomycin and phage EFLK1 showed massive bacterial lysis and degradation, leaving almost no trace of the biofilm (Figure 6, panel D).

### 3.3. Identifying Cell Wall Changes Followed Phage Treatment

**Figure 7:**
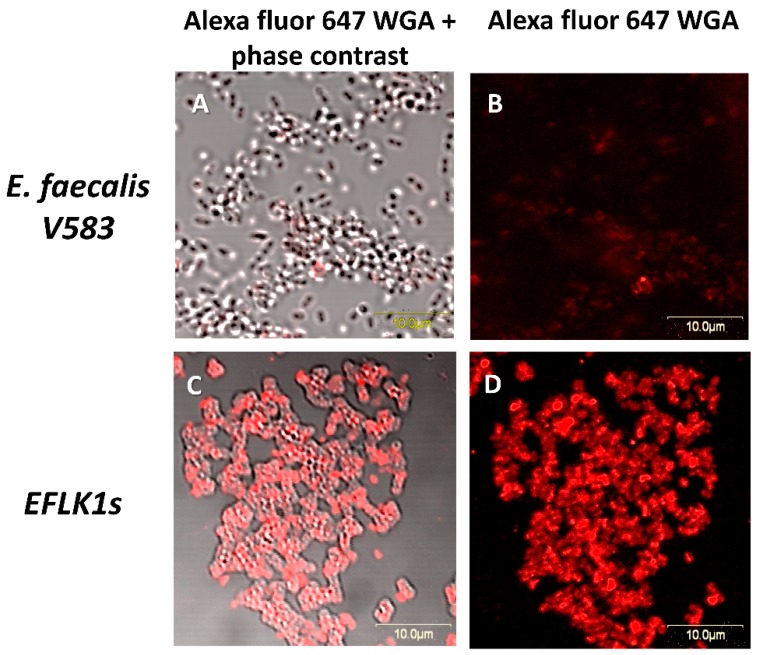
Alexa Fluor 647 wheat germ agglutinin (WGA) stains *EFLK1 survivors*. Confocal microscopy images (MAG: 60): (**A**–**B**) vancomycin resistant *E. faecalis* V583 show low fluorescence level, indicating no conjugation to *N*-acetylglucosamine. (**C**–**D**) *EFLK1 survivors* show high fluorescence levels followed the conjugation of Alexa Fluor 647 WGA to *N*-acetylglucosamine.

**Figure 8:**
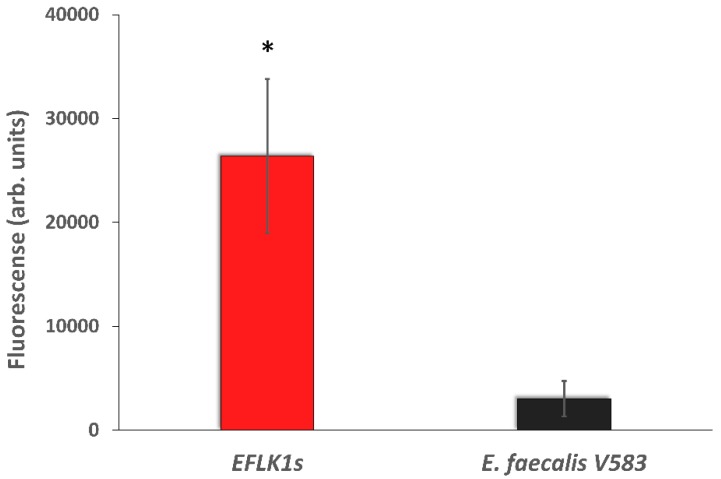
Fluorescence activated cell sorting (FACS) analysis: higher fluorescence levels are obtained followed phage treatment. Red = *EFLK1* survivors, black = untreated control of *E. faecalis* V583. Statistically significant (*p* < 0.01) compared to the untreated control. The results are mean ± SD based on six independent biological replicates.

Since both phages and vancomycin target bacterial cell wall we decided to further explore their synergistic mechanism by detecting *N*-acetylglucosamine after EFLK1 phage treatment. We found that the ability of the lectin to bind cell wall was changed after phage treatment, as can be seen in Figure 7. The fluorescence staining of Alexa Fluor 647 WGA increased 8-fold following phage treatment (*EFLK1s*), suggesting a change in the peptidoglycan of the cell wall (Figure 8).

## 4. Discussion

This study addresses two key healthcare concerns: the lack of effective biofilm treatment options and the issue of antibiotic-resistant pathogens. Specifically, we tested whether phage-antibiotic synergy (PAS) also occurs when bacteria are considered resistant to the antibiotic. We demonstrated that in a model of VRE *faecalis* treated with a combination of vancomycin and an *E. faecalis*-specific phage, the antibiotic that alone had almost no effect induced phage lethality. The combined treatment effectively targeted planktonic and biofilm associated VRE. As expected, when used alone, only extremely high concentrations (>0.25 mg/mL) of the antibiotic eradicated the infection. However, when the phage was combined with the antibiotic, much lower (0.015 mg/mL) antibiotic concentrations demonstrated significant antibacterial effects.

Vancomycin, a last-resort antibiotic, is used mainly in serious Gram-positive bacterial infections that do not respond to other antibiotics. This effective antibiotic inhibits cell wall synthesis in Gram-positive bacteria but is ineffective against VRE. The present study showed that for the same amount of *E. faecalis* killing, less vancomycin was required in the presence of phage. Moreover, VRE growth and viability were reduced not only in planktonic cultures but also in biofilm cultures following exposure to combinations of the two. Combining vancomycin with phage EFLK1 produced a synergistic effect, resulting in almost no resistant bacteria surviving in some of the treatment combinations (Figure 3, Figure 4 and Figure 5). Although the phage was previously shown to be effective against VRE [10,35], especially as a phage cocktail [10], this effect was intensified when the treatment modality included vancomycin, an antibiotic to which the target bacteria are resistant. Although this effect was present in other *E. faecalis* strains (Table 1), it was highly efficacious when targeting *E. faecalis* V583.

Treatments involving combinations of phages and antibiotics were successfully practiced in the 1950s and 1960s in Soviet medicine [39,40,41,42]. Several mechanisms were previously suggested for the PAS effect. The most studied is phage λ induction by SOS-inducing agents [43]. In the case of β-lactam antibiotics against *E. coli,* PAS is suggested to be a consequence of cellular filamentation unrelated to the SOS system, which inhibits bacterial cell division in response to DNA damage [24]. This effect has been achieved with various phages against different pathogens; thus, the presence of antibiotic is thought to render an advantage to phages [24]. Similarly, here, exposing VRE to both phage EFLK1 and vancomycin resulted in a greater killing effect, whereas vancomycin alone only minimally affected the bacteria. However, our case appears to be unique, as vancomycin is the antibiotic to which these bacteria are resistant. Phage EFLK1 exerted broad antibacterial activity against clinical isolates, making it a valuable treatment option for *E. faecalis*-related infections (Table 1).

Biofilm-based infections are extremely difficult to target [38]. Nevertheless, combining phage EFLK1 with vancomycin also reduced viable counts by nearly eight log and the biomass by 87% in a well-established biofilm (Figure 4 and Figure 5), whereas vancomycin alone failed entirely. Treating biofilm with an efficient phage, such as phage EFLK1, showed good results (four log growth reduction), and combining phage EFLK1 with an antibiotic led to much better results. Surprisingly, the lower vancomycin dose showed a better antibacterial effect when vancomycin was combined with the phage. A possible explanation may be found in the previously described information that sublethal concentrations of certain antibiotics stimulate the host bacteria to produce virulent phages by increasing the bacterial cell biomass and accelerating lysis of the infected host cells, inducing the phages to spread faster [24]. Ryan et al. found that combining T4 bacteriophages and cefotaxime significantly enhanced the eradication of bacterial biofilms compared with cefotaxime treatment alone [25], reducing the minimum biofilm eradication concentration of cefotaxime against *E. coli* biofilms. Another study showed synergism between ciprofloxacin, meropenem, tetracycline, and KS12 and KS14 phages in *B. cenocepacia*, strains C6433 and K56-2, which was reflected in their enlarged plaque size [27]. Phages and penicillin have also been successfully combined against *Staphylococcus* [28,29]. Huff et al. observed a similar phenomenon using *E. coli* phages with ciprofloxacin [44]. Hagens et al. [45] suggested that the bacterial outer membrane was ineffective as a barrier against antibiotic penetration into the bacterial cell during filamentous phage progeny extrusion regarding phages released from the bacteria by extrusion rather than by cell lysis [29]. We found that the combined effect of phages and antibiotics can be useful for treating bacteria within the biofilm matrix and that the combination synergistically enhanced biofilm eradication. In vivo studies in mice explored combining phages and antibiotics such as dichlortetracycline, erythromycin, pasomycin and oxacillin. The best results for these combinations were achieved when the antibiotic was administered 24 h before phage treatment [42].

Interestingly, when phage EFLK1 was inoculated with VRE at higher multiplicity of infection (MOI), the viable counts (CFU/mL) of the treated biofilm increased. This phenomenon was previously reported by Cheng et al. [46], speculating that when phage EF-P29 is incubated with a *VRE faecalis* strain at a higher MOI, phage-resistance mutations in *E. faecalis* occur quickly. It has been suggested that a high MOI enhances the selection towards phage resistance, while a low MOI allows the phage-sensitive bacteria to persist and outcompete the phage-resistant bacteria [46,47]. This phage resistance development is one of the therapeutic concerns of phage therapy [48]. However, the positive anti-VRE effect of phages and antibiotics shown here indicates that the combined approach may be an optional solution to delay the appearance of phage-resistant variants and enhance treatment efficacy [47]. Moreover, for resistant bacteria such as methicillin-resistant *Staphylococcus aureus* (MRSA), the recent guidelines include the use of 0.015–0.020 mg/mL of vancomycin [49]. Our results show that although vancomycin-resistant bacteria can be targeted only at > 0.25 mg/mL, the combination of vancomycin at a concentration of 0.015 mg/mL and low phage EFLK1 MOI resulted in efficient antibacterial effects against VRE. This combination may suggest an optional solution to overcome both phage resistance and antibiotic resistance concerns.

Consistent with previous findings, SEM micrographs of biofilms exposed to phage EFLK1 revealed extensive bacterial lysis, leaving mainly the extracellular matrix. This effect was amplified when the VRE biofilm was exposed to vancomycin and phage EFLK1, showing massive bacterial lysis, degradation and biofilm deformation, leaving almost no trace of the biofilm (Figure 6).

The exact mechanism of the synergistic effect between phage EFLK1 and vancomycin is being intensively investigated in our lab. Since the presence of bacteriophages may increase bacterial sensitivity to antibiotics, the mechanism behind the PAS phenomenon observed here may be related to the phage and antibiotic binding sites. Vancomycin inhibits cell wall synthesis in Gram-positive bacteria by targeting the D-alanyl-D-alanine (D-Ala-D-Ala) terminus of the intermediates in peptidoglycan synthesis. Thus, vancomycin inhibits the transglycosylation and transpeptidation reactions in peptidoglycan assembly [50]. Binding of fluorescently labelled wheat germ agglutinin (WGA), a lectin that specifically binds terminal GlcNAc residues in wall teichoic acids may indicate changes in cell wall glycosylation [37]. Alexa Fluor 647 WGA was able to stain phage treated bacteria, but this lectin almost failed to bind the untreated bacteria (Figure 7 and Figure 8), pointing at a lack of GlcNAc residues in wall teichoic acids. This may increase the bacterial sensitivity to vancomycin and may explain the lower vancomycin MIC of the phage treated bacteria. The *E. faecalis* mechanism of vancomycin resistance is due to an alternative cell wall precursor production pathway that poorly binds vancomycin [51]. We suggest that binding of the phage to the cell wall is likely related to enzymes that alter the terminal peptidoglycan residue so that when the bacteria are exposed to the phage, their enzymatic action is blocked, which induces bacterial sensitivity to vancomycin. 

Another possible explanation is based on the phage endolysins. Endolysins were shown to have a direct activity against enzymes that are responsible for covalent linkages in the bacterial cell wall, such as glycosylase, trans-glycosylase, amidase and endopeptidase [52]. The synergistic activity may be a result of a simultaneous anti enzymatic activity. (Lipo)teichoic acids embedded in peptidoglycan are an example of essential component of Gram-positive bacteria that are often a primary phage receptor. Polysaccharide depolymerases and virion-associated lysins (VALs) are carbohydrate active enzymes that recognize, bind and degrade bacterial polysaccharide to gain access to a secondary receptor on the bacterial cell surface [53]. The primary receptor within the peptidoglycan to which the phage or its enzymes bind may be related to vancomycin target. Further study is needed to investigate the presence and function of enzymes such as VALs in order to establish the mechanism of action by which vancomycin sensitivity is reversed. Additionally, using different antibiotics combined with our phage against VRE has potential and merits further investigation.

In conclusion, a synergistic antibacterial effect was obtained using phage EFLK1 with antibiotics, even in cases of resistance to certain antibiotics such as vancomycin. Our results support the idea that antibiotic resistance can be attenuated, and potentially, when combined with phage therapy, we may again successfully use the antibiotics to which pathogens have developed resistance. This may significantly impact future development of treatment modalities against antibiotic-resistant pathogens. Further study of the PAS mechanism of vancomycin and phages against VRE is required.

## Figures and Tables

**Table 1:** Bacterial strains and their sensitivities to phage EFLK1, vancomycin and combined treatment.

Bacterial Strain	Origin ^a^	Antibiotic Resistance ^b^	Phage EFLK1 ^c^	Vancomycin MIC (mg/mL) ^d^	Phage EFLK1 + Vancomycin MIC (mg/mL) ^e^
*Enterococcus faecalis* V583	ATCC 700802	Vancomycin Gentamicin	S	>0.25	0.015
*Enterococcus faecalis* Aef01	Clinically isolated from urine	-	S	0.003	<0.001
*Enterococcus faecalis* Aef03	Clinically isolated from urine	-	S	0.007	<0.001
*Enterococcus faecalis* Aef05	Clinically isolated from venal blood flow	Erythromycin Gentamicin	S	0.003	<0.001

^a^ The clinical isolates were performed at the Hadassah Medical Center, Jerusalem, Israel. ^b^ Bacterial resistance of the clinical isolates to antibiotics was determined by the infectious disease unit of Hadassah Hospital, Jerusalem, Israel. ^c^ Bacterial sensitivity of the clinical isolates to phage EFLK1 (1 × 10^9^ plaque forming units (PFU)/mL): S, sensitive. ^d^ Strains were grown in a 96-well plate reader for 24 h. Vancomycin hydrochloride (0.25 mg/mL to 0.001 mg/mL) was added at time zero, and the optical density was recorded every 20 min. ^e^ Strains were grown in a 96-well plate reader for 24 h. Phage EFLK1 (multiplicity of infection (MOI) of 10^3^) and vancomycin hydrochloride (0.25 mg/mL to 0.001 mg/mL) were added at time zero, and the optical density was recorded every 20 min.

## Supplementary Materials

The following are available online at https://www.mdpi.com/1999-4915/11/10/954/s1, Figure S1: Scheme of the experimental setup in a 96-well microtiter plate, Figure S2: Viable counts of all the combined treatments of phage EFLK1 and vancomycin against VRE planktonic cells and Figure S3: Viable counts of all the combined treatments of phage EFLK1 and vancomycin against VRE 72 h biofilm.
