# Supplementary material for: Eradication of Vancomycin-Resistant Enterococci by Combining Phage and Vancomycin
Source: Viruses. 2019 Oct 16;11(10):954. doi: 10.3390/v11100954 (PMC6833023; doi:10.3390/v11100954)
Supplement: Supplementary file 1 [file viruses-11-00954-s001.pdf]

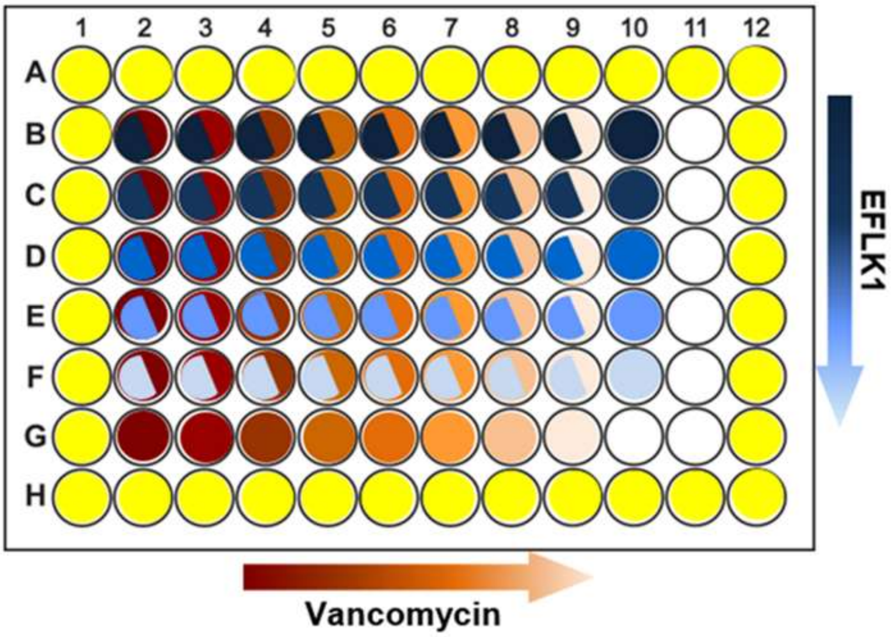

.

Figure S1. Scheme of the experimental setup in a 96-well microtiter plate. *E. faecalis* was treated with twofold serial dilutions of phage EFLK1 in BHI broth (columns, marked blue; from 1 × 10^9 PFU/well phage EFLK1 to 3.1 × 10^7 PFU/well phage EFLK1). *E. faecalis* was treated with twofold serial dilutions of vancomycin hydrochloride in BHI broth (rows, marked red: from 0.25 mg/mL to 0.001 mg/mL). The growth kinetics of the cultures were recorded (OD_{600}) at 37 °C shaking for 5 s every 20 min in a 96-well plate reader (Synergy; BioTek, Winooski, VT, USA). Samples were plated at the endpoint and incubated for 24 h at 37 °C. Viable counts were determined at the endpoint by counting CFU/mL. Blue = phage EFLK1, Red = vancomycin, Blue and red combinations = vancomycin combined with phage EFLK1. White = wells containing *E. faecalis*. Yellow = wells containing DDW (to prevent an edge effect).

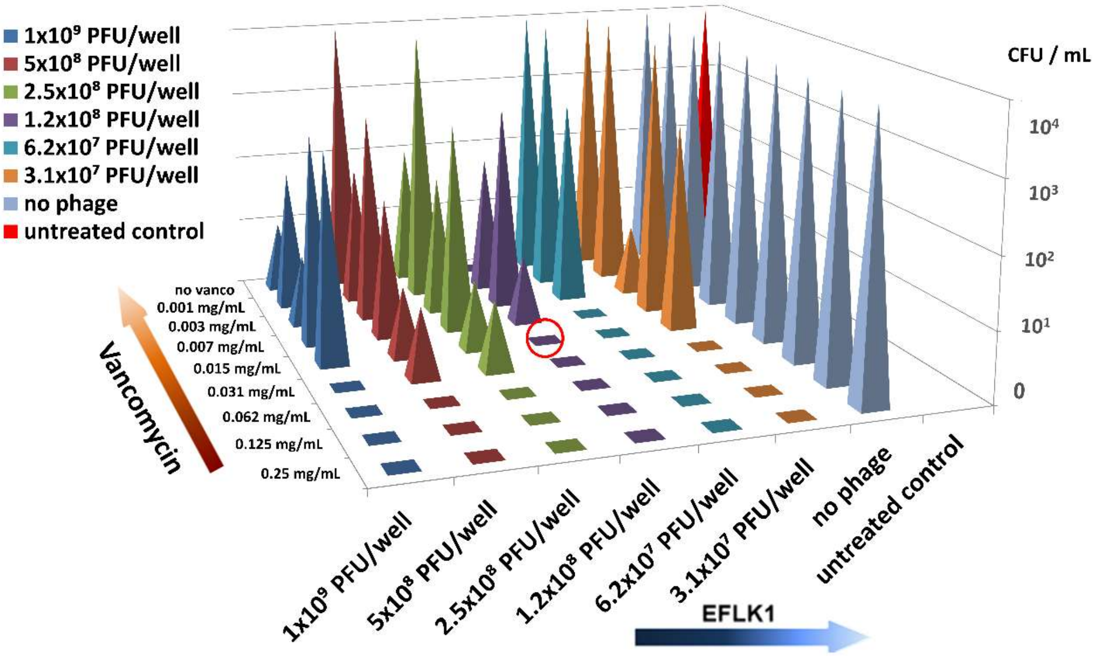

Figure S2. Viable counts of all the combined treatments of phage EFLK1 and vancomycin against VRE planktonic cells. After 24-h treatment of exponential bacterial growth with 1×10^9 to 3.1×10^7 PFU/well phage EFLK1 combined with 0.25 to 0.001 mg/mL vancomycin five microliters from each well were plated on BHI. Colonies were counted after 24 h at 37 °C. Maximal bacterial growth was observed in the untreated VRE controls (red) and in the vancomycin treated cells (light blue), and minimal growth was seen in combinations of vancomycin > 0.015 mg/mL and phage EFLK1 > 3.1×10^7 PFU/well. Phage EFLK1 alone reduced the bacterial growth, although the inhibition was less efficient than when in combination with vancomycin. The mixture of the lowest treatment concentrations that yielded the best synergistic effect and affected both planktonic and biofilm was 0.015 mg/mL vancomycin and 1.2×10^8 PFU/well phage EFLK1 (red circle).

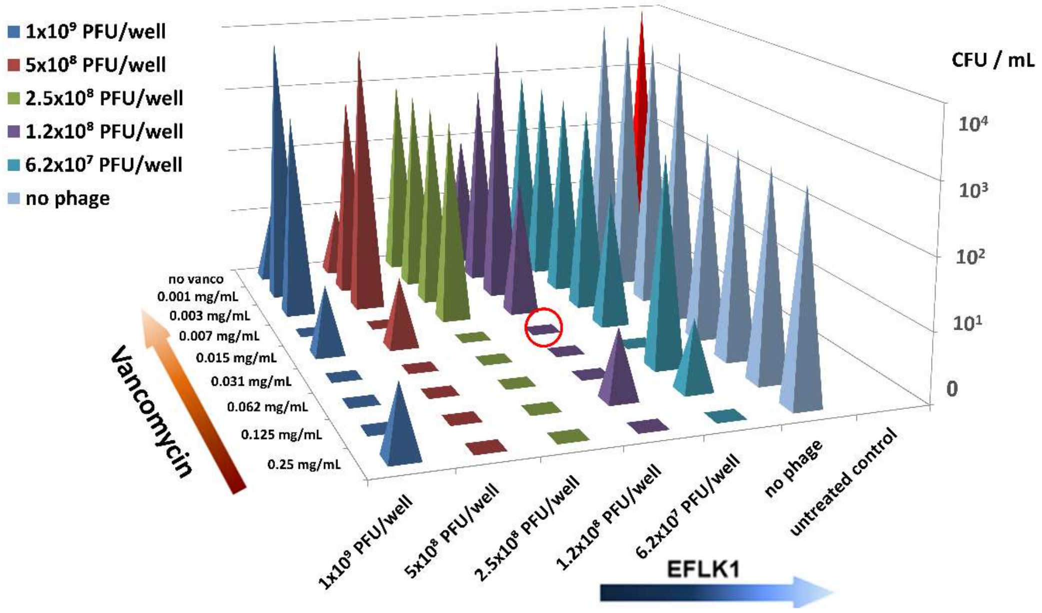

Figure S3. Viable counts of all the combined treatments of phage EFLK1 and vancomycin against VRE 72-h biofilm. After 96 h of treatment with 1×10^9 to 6.2×10^7 PFU/well phage EFLK1 combined with 0.25 to 0.001 mg/mL vancomycin, five microliters from each well were plated on BHI. Colonies were counted after 24 h at 37 °C. Maximal bacterial growth was observed in the untreated VRE controls (red) and in the vancomycin treated cells (light blue), and minimal growth was seen in combinations of vancomycin > 0.007 mg/mL and phage EFLK1 > 6.2×10^7 PFU/well. Phage EFLK1 alone reduced the bacterial growth at all concentrations, although the inhibition was less efficient than when in combination with vancomycin. The mixture of the lowest treatment concentrations that yielded the best synergistic effect and affected both planktonic and biofilm was 0.015 mg/mL vancomycin and 1.2×10^8 PFU/well phage EFLK1 (red circle).
